# *Bordetella bronchiseptica* Colonization Limits Efficacy, but Not Immunogenicity, of Live-Attenuated Influenza Virus Vaccine and Enhances Pathogenesis After Influenza Challenge

**DOI:** 10.3389/fimmu.2018.02255

**Published:** 2018-10-04

**Authors:** Holly R. Hughes, Susan L. Brockmeier, Crystal L. Loving

**Affiliations:** ^1^Virus and Prion Diseases of Livestock Research Unit, National Animal Disease Center, Agricultural Research Services, U.S. Department of Agriculture, Ames, IA, United States; ^2^Food Safety and Enteric Pathogens Research Unit, National Animal Disease Center, Agricultural Research Services, U.S. Department of Agriculture, Ames, IA, United States

**Keywords:** influenza vaccine, vaccine, co-infection, efficacy, immunity

## Abstract

Intranasally administered live-attenuated influenza virus (LAIV) vaccines provide significant protection against heterologous influenza A virus (IAV) challenge. However, LAIV administration can modify the bacterial microbiota in the upper respiratory tract, including alterations in species that cause pneumonia. We sought to evaluate the effect of *Bordetella bronchiseptica* colonization on LAIV immunogenicity and efficacy in swine, and the impact of LAIV and IAV challenge on *B. bronchiseptica* colonization and disease. LAIV immunogenicity was not significantly impacted by *B. bronchiseptica* colonization, but protective efficacy against heterologous IAV challenge in the upper respiratory tract was impaired. Titers of IAV in the nose and trachea of pigs that received LAIV were significantly reduced when compared to non-vaccinated, challenged controls, regardless of *B. bronchiseptica* infection. Pneumonia scores were higher in pigs colonized with *B. bronchiseptica* and challenged with IAV, but this was regardless of LAIV vaccination status. While LAIV vaccination provided significant protection against heterologous IAV challenge, the protection was not sterilizing and IAV replicated in the respiratory tract of all LAIV vaccinated pig. The interaction between IAV, *B. bronchiseptica*, and host led to development of acute-type B. bronchiseptica lesions in the lung. Thus, the data presented do not negate the efficacy of LAIV vaccination, but instead indicate that controlling *B. bronchiseptica* colonization in swine could limit the negative interaction between IAV and *Bordetella* on swine health.

## Introduction

Influenza A virus (IAV) is a major animal and public health concern given the zoonotic nature of IAV (1–3). As a natural host to IAV, research on IAV in swine has relevance to both human and animal medicine. IAV is a segmented, negative-sense, single-stranded RNA virus. The surface glycoproteins, hemaggluttinin (HA) and neuraminidase (NA), are used to type IAV, and currently H1N1, H1N2, and H3N2 viruses circulate in pigs in the United States (4, 5). There are a large number of IAV H1 and H3 genetic and antigenic variants co-circulating, and continued antigenic drift and shift of circulating viruses has made control of IAV in swine very difficult (5). Live-attenuated influenza virus (LAIV) vaccination in swine has been shown to provide cross-protection against heterologous IAV of the same subtype, and partial protection against different subtypes [reviewed in Sandbulte et al. (6)]. LAIV is licensed for use in humans and was recently approved for use in swine, with numerous experimental studies documenting improved efficacy of LAIV over inactivated vaccines (7, 8). Several LAIV vaccines for use in swine have been developed; each with a different attenuation mechanism (9–11). Similar to humans, intranasal LAIV vaccination in pigs induces the production of IAV-specific mucosal IgA, but little peripheral IAV-specific IgG (8). The induction of immunity in the respiratory tract has been shown to be the mechanism by which LAIV vaccines provide significant cross-protection against heterologous strains of IAV, limiting viral replication throughout the respiratory tract [reviewed in Rose et al. (12)].

*Bordetella bronchiseptica* can colonize the respiratory tract of a large number of mammals, including mice, rabbits, dogs and pigs, among others. Respiratory disease associated with *B. bronchiseptica* covers a wide spectrum, including kennel cough in dogs and atrophic rhinitis in pigs (13, 14). In humans, *B. pertussis* infection can lead to whooping cough, though colonization without clinical presentation has been documented (15, 16). Similarly, *B. bronchiseptica* exposure to pigs can result in chronic, asymptomatic colonization of the respiratory tract and it is believed to be ubiquitous in swine production systems. Co-infection with IAV or coronavirus and *B. bronchiseptica* in pigs causes exacerbated pulmonary disease, indicating the negative impact of *B. bronchiseptica* colonization with viral infection (17, 18). *Bordetella* species encode for a number of virulence factors, including tracheal cytotoxin, dermonecrotic toxin, lipopolysaccharide, and a type III secretion system (19). While the gene locus controlling expression of many virulence factors, including the type III secretion system, has been highly investigated, factors that alter expression of virulence genes *in vivo* are not completely understood (20, 21).

In the past decade, the complex interaction between mucosal surfaces and colonizing microbiota has been recognized as important in modulating both health and disease states [reviewed in Esposito and principi (22)]. The commensal microbiota of the upper respiratory tract includes bacterial species in which colonization alone does not lead to clinical disease, but upon a stressful event (i.e., viral infection, immunosuppression) these bacteria play a major role in disease pathogenesis, often referred to as pathobionts. Administration of LAIV vaccine induces changes in the nasal microbiota and gene expression in nasal epithelium. In addition, LAIV administration alters colonization dynamics of important bacterial pathogens (23). Given the ubiquitous nature of *B. bronchiseptica* in swine and the documented increase in disease following *B. bronchiseptica* with IAV co-infection, we performed a study to determine if *B. bronchiseptica* colonization prior to LAIV vaccination altered LAIV immunogenicity and efficacy against heterologous IAV challenge, or the dynamics of *B. bronchiseptica* colonization.

## Materials and methods

### Influenza viruses and *B. bronchiseptica* inocula

*B. bronchiseptica* strain KM22 is a virulent phase I swine isolate initially cultured from a herd with atrophic rhinitis, and has been used extensively by our group for studies (17, 18, 24–26)*. B. bronchiseptica* inoculum was prepared as previously described (18). Pigs were inoculated intranasally (IN) with 1 ml (0.5 ml/nostril) of the final inoculum, which was confirmed to be 6(log_10_) CFU/ml. LAIV vaccine was prepared and used as previously described (8). The LAIV used encoded for surface genes HA and NA from pandemic influenza (H1N1, A/NY/18/2009) and internal genes from A/Turkey/Ohio/313053/2004, which is a swine-like virus (generously provided by Dr. Daniel Perez, University of Georgia) with a truncation in the NS1 gene for attenuation. A heterologous β-cluster H1N2 swine IAV isolate (A/swine/Minnesota/03012/2010; MN10) was used as challenge virus. Both LAIV and challenge virus was propagated in Madine-Darby Canine Kidney (MDCK) cells. The LAIV inoculum titered at 5.5 (log_10_) TCID_50_/ml and the MN10 challenge virus titered at 5.8 (log_10_) TCID_50_/ml. Pigs received 2 ml of LAIV or challenge virus at each indicated inoculation dates.

### Experimental design

Animal studies were conducted in accordance with the National Animal Disease Center's Institutional Animal Care and Use Committee. Pregnant sows were transferred to the National Animal Disease Center (NADC) approximately 2 weeks prior to their farrowing due date from a herd negative for IAV-specific antibody and porcine reproductive and respiratory syndrome virus. Piglets were early weaned from sows at approximately 10 days of age to reduce vertical transmission of bacterial respiratory pathogens colonizing the sow to the piglets. Piglets were transferred to individual isolation rooms based on treatment, with piglets from each litter represented in each treatment group (Table 1). Nasal swabs were collected from piglets prior to any inoculation and neither IAV nor *B. bronchiseptica* were isolated. At 2 weeks of age, piglets in groups 4-6 were inoculated with *B. bronchiseptica* (Bb) by the intranasal (IN) route, and this was considered day−7 of the experiment. Seven days later when piglets were 3 weeks of age, animals in groups 3 and 6 were inoculated with LAIV by the IN route and this was considered day 0 of the study. A second dose of LAIV was administered on study day 21 (3 weeks post-LAIV priming). Nasal swabs were collected on day−7 (Bb inoculation), day 0 (immediately prior to LAIV administration) and days 1–3 for evaluation of Bb nasal colonization following LAIV vaccination. Nasal washes were collected on days 7, 21 (immediately prior to LAIV boost), 28 (1 week post-boost), and 42 for evaluation of Bb nasal colonization and IAV-specific mucosal antibody titers. Blood was collected on day−7 and 42 for serum isolation to evaluate antibody titers. On day 42, pigs in groups 2, 3, 5, and 6 were challenged with heterologous IAV by the IN route. Nasal swabs were collected every day, for 5 days, following IAV challenge for evaluation of IAV titers in the nose. On day post-infection (dpi) 5, pigs were euthanized with a lethal dose of pentobarbital and necropsy performed.

**Table 1:** Experimental groups and treatments in the study.

**Group #**	**Group^a^ designation**	***N*^b^**	**Bb**	**LAIV**	**IAV challenge**
1	Control	7	No	No	No
2	NV/Ch	12	No	No	Yes
3	LAIV/Ch	12	No	Yes	Yes
4	Bb/NV/NCh	6	Yes	No	No
5	Bb/NV/Ch	7	Yes	No	Yes
6	Bb/LAIV/Ch	8	Yes	Yes	Yes

a *NV, Non-Vaccinated; Ch, Challenged; LAIV, Live-Attenuated Influenza Virus; Bb, B. bronchiseptica; NCh, Non-Challenged*.

b *N, number of pigs in indicated group*.

At necropsy, gross lung lesion scores were determined based on the percentage of each lung lobe affected and the percentage of total lung volume each lobe represented, calculated as initially described (27). Postmortem samples collected included nasal swab, trachea wash, and broncho-alveolar lung lavage fluid (BALF). Nasal swab, nasal wash and trachea wash samples were collected as previously described (8, 18), with minor modifications. Briefly, nasal swabs were collected in 2 ml of minimal essential media (MEM) and trachea wash was performed in 3 ml MEM. BALF was collected by lavaging all lobes with 50 ml of MEM and recovering as much as possible, approximately 15-20 ml. Sections of lung were collected for microscopic evaluation. Lung tissues were fixed in 10% neutral buffered formalin for 72 h, and then processed and stained with hematoxylin and eosin using routine procedures.

### Mucosal antibody evaluation

Nasal wash samples collected on the day of IAV challenge (day 42) were used in an indirect, whole-virus enzyme-linked immunosorbent assay (ELISA) as previously described (7, 8). Briefly, concentrated vaccine virus or challenge virus was used as antigen (15 μg/ml; 0.05 ml per well) and nasal wash was used as sample in the assay. IAV-specific IgA was detected with horseradish peroxidase-labeled anti-swine IgA (1:2,000 dilution; clone Bethyl Laboratories). IAV-specific IgA endpoint titers were determined by titrating samples two-fold in duplicate before performing the ELISA. The optical density (OD) data was modeled as a nonlinear function of the Log_10_ dilution using GraphPad Prism 6 (GraphPad software Inc, La Jolla, CA) log (agonist) vs. response-variable slope four-parameter logistic model. Endpoints were interpolated by using 2X the average OD of the nasal wash samples from non-vaccinated controls as the cutoff.

### IAV and *B. bronchiseptica* isolation

Number of colony-forming units (CFU) of *B. bronchiseptica* per ml of swab fluid or nasal wash, tracheal wash, and BALF were determined as previously described (18, 25). Briefly, samples were serially diluted in phosphate-buffered saline (PBS) and plated on blood agar (trachea wash and BALF) or blood agar supplemented with 2 ug/ml amikacin, 4 ug/ml vancomycin, and 4 ug/ml amphotericin B (nasal swab and nasal wash). An aliquot of BALF was also plated on brain-heart infusion agar supplemented with 0.01 % NAD (w/v) and 5 % horse serum to rule out aerobic bacterial infection (other than *B. bronchiseptica* in respective groups). For determining viral load, nasal swab, trachea wash and BALF were thawed, filtered with a 0.45 um syringe filter, and fluid was used as previously described for virus isolation and titration (7), with immunohistochemistry staining used for evaluation (8) and the log10-transformed number of TCID_50_/ml of each sample calculated by the method of Reed and Muench (28). Samples that were negative by virus isolation were assigned a value of zero. Samples that were negative on virus titration but positive by virus isolation were assigned a value of 0.5 (log_10_) TCID_50_/ml.

BALF was collected at necropsy (dpi 5) and an aliquot centrifuged at 500 x g for 10 min. The cell-free supernatant was collected and used to evaluate cytokine levels in the lung. The amount of CCL2 (MCP-1), and IFN-α, IL-8, IL-1β, IL-10, and IFN-γ in the cell-free lung lavage was determined by single-plex (KingFisher Biotech) or multiplex cytokine ELISA (Aushon Biosystems), respectively, as previously described (29).

### Data analysis

A two-factor repeated measures mixed effects model analysis of Log (CFU) differences between the treatment groups Bb/LAIV and Bb/NV and a dpi repeated measures factor using pigs as subjects was used to evaluate statistical differences in Bb nasal colonization (SAS, v9.2). A weighted regression equation of Log (TCID_50_) as a function of dpi for Bb/LAIV/Ch, Bb/NV/Ch, LAIV/Ch, and NV/Ch groups using mean Log (TCID_50_) values and standard weights of 1/variance with 95% confidence limits was used for statistical comparisons of nasal shedding (SAS, v9.2). A one-way ANOVA with a Tukey's post-test was used to evaluate differences in lung lesions, respiratory tract Bb colonization, and respiratory tract IAV burden (GraphPad Prism, v6). An unpaired students *t*-test was used for analysis of IAV-specific IgA levels, and a Kruskal-Wallis test with a Dunn's multiple comparisons post-test was used to determine statistical differences in lung cytokine levels (GraphPad Prism, v6).

## Results

### LAIV administration had minimal impact on *B. bronchiseptica* nasal colonization

**Figure 1:**
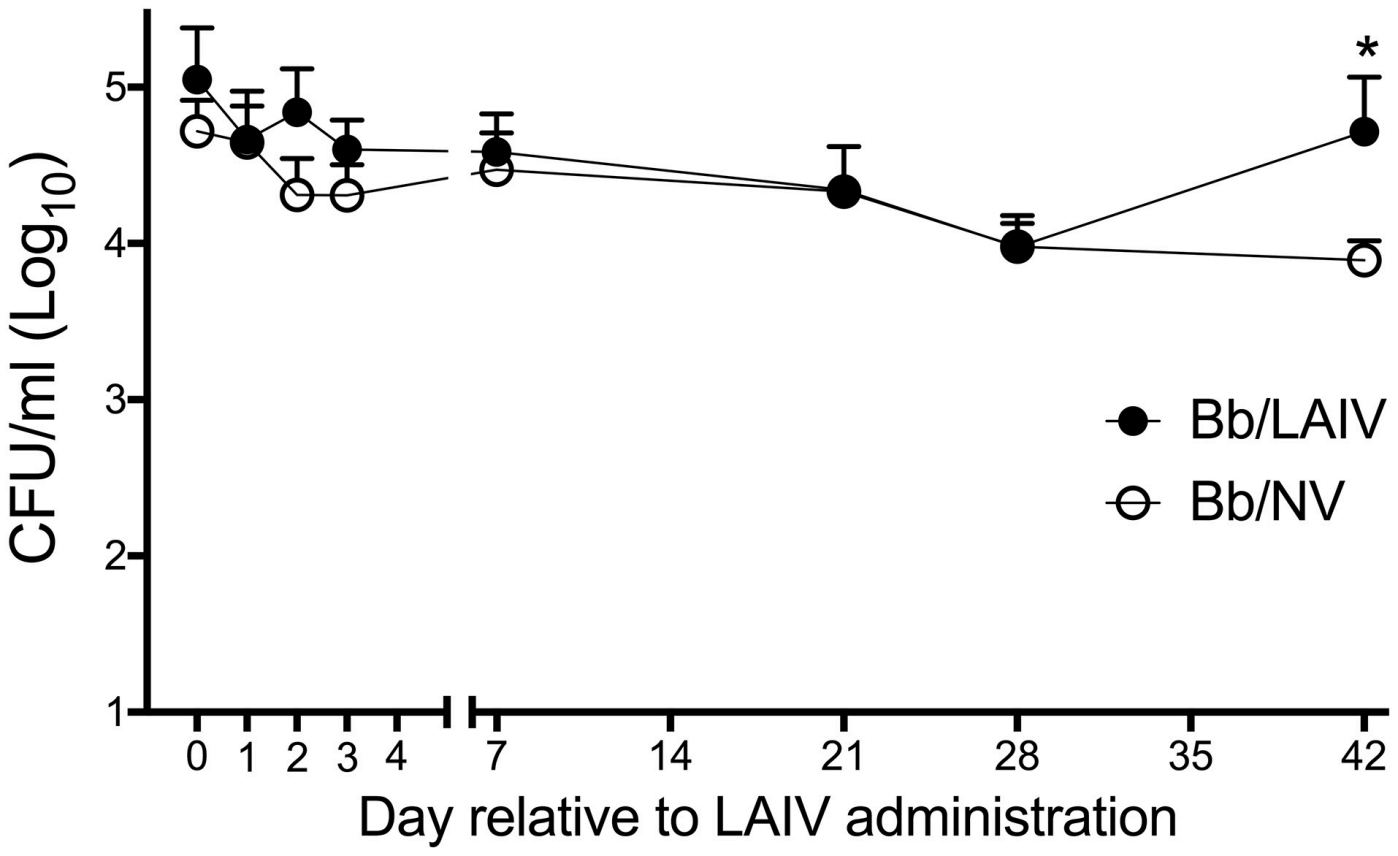
*B. bronchiseptica* nasal colonization following intranasal LAIV administration. To determine if *B. bronchiseptica* (Bb) nasal colonization was impacted by LAIV administration, Bb CFU's were enumerated at indicated day's post-LAIV administration. Pigs were intranasally inoculated with Bb on day−7 (not shown) and LAIV on days 0 and 21. In accordance with Table 1, pigs in groups 4 and 5 were included in the Bb/NV group (*n* = 13) and group 6 pigs were used for Bb/LAIV group (*n* = 8). Data is reported as the number of CFU per ml in nasal swabs (days 0–3) and nasal wash (days 7, 21, 28, and 42). Significant differences (*p* < 0.05) in Bb CFU are noted with an asterisk (*).

Given that the LAIV vaccine was delivered by the IN route, the effect of LAIV administration on *B. bronchiseptica* nasal colonization was assessed at various time points following LAIV vaccination. At the time the initial dose of LAIV was administered, which was 1 week after *B. bronchiseptica* inoculation, there was no significant difference in *B. bronchiseptica* colonization between treatment groups (*p* > 0.05; Figure 1). While there was a numerical trend for differences between groups at days 2 and 3 following the initial dose of LAIV, there was not a statistical difference between groups (*p* > 0.05). Pigs in the LAIV group were boosted at day 21, and again, there was not a significant difference in *B. bronchiseptica* colonization between treatment groups at the time of LAIV administration (day 21), nor 1 week following administration of the booster dose (day 28, *p* > 0.05). Only on day 42 relative to LAIV administration was there a significant difference (*p* < 0.05) in *B. bronchiseptica* CFUs in nasal wash between Bb/LAIV and Bb/NV treatment groups, though on average, it was limited to less than a log_10_ difference (4.72 ± 0.92 vs. 3.89 ± 0.43, log_10_ CFU ± SEM/ml respectively).

### Pneumonia was more severe in pigs colonized with *B. bronchiseptica* and challenged with heterologous IAV, regardless of LAIV vaccination status

**Figure 2:**
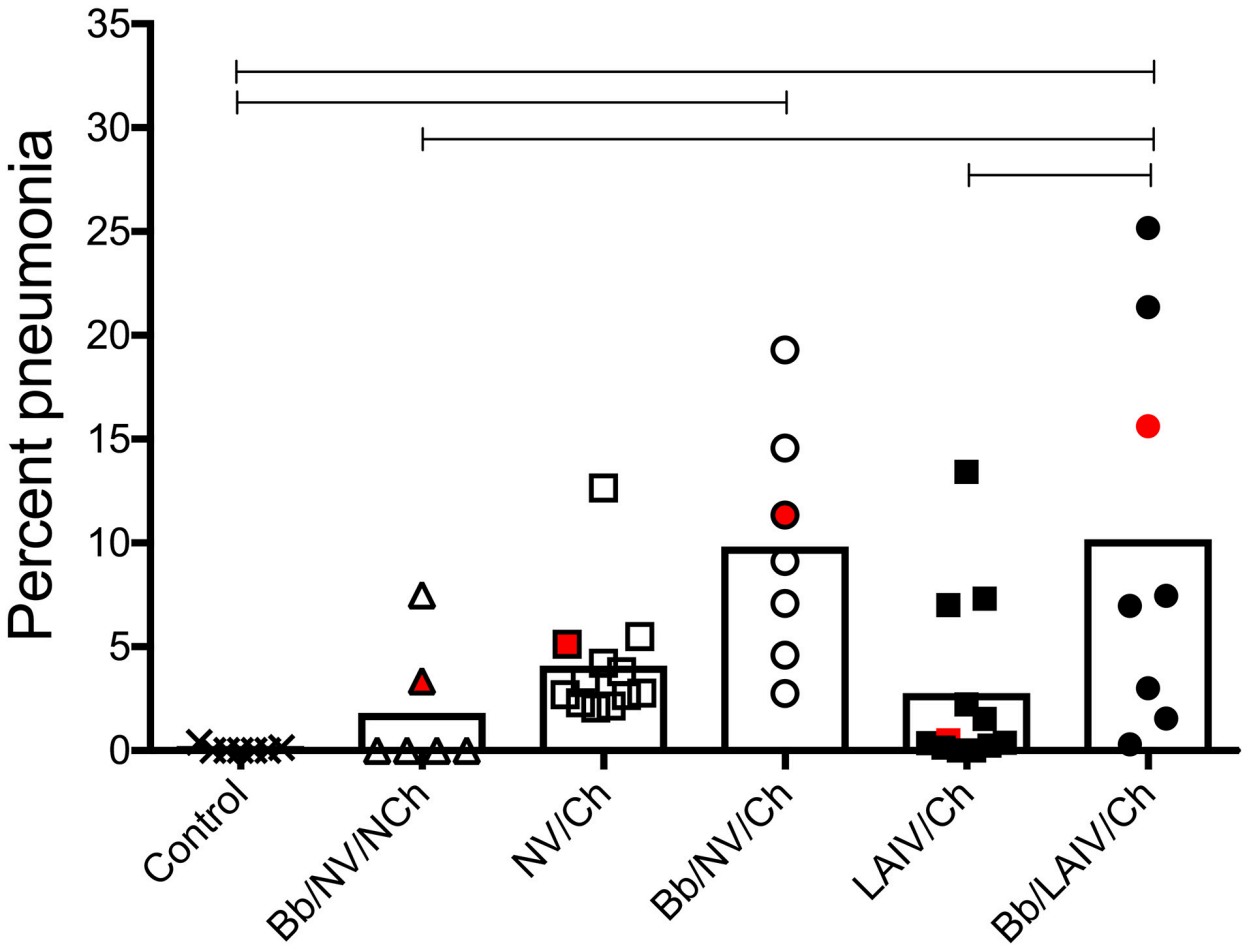
Severity of pneumonia was associated with IAV challenge in *B. bronchiseptica* colonized pigs, regardless of LAIV vaccine status. Groups of pigs were inoculated with *B. bronchiseptica* (Bb) on day−7, LAIV on days 0 and 21, and subsequently challenged (Ch) with heterologous IAV as described in materials and methods. A Non-vaccinated (NV) group and a NV/non-challenged (NV/NCh) control group were included as controls. At 5 days post-infection, the percentage of lung affected with macroscopic lesions was determined and reported as percent pneumonia, calculated as described in materials and methods. Each dot represents the score for a pig in that respective group, with averages indicated by bars. The red data point indicates the score and sample of lung section used for Figure 3. Data was analyzed using a one-way ANOVA with a Tukey's post-test (GraphPad Prism 6). Groups with connecting lines were significantly different (*p* < 0.05).

Macroscopic pneumonia in the strict control group (non-infected, non-vaccinated) was minimal (0.08 ± 0.15), and macroscopic lesions were detected in only 2 of the 6 pigs inoculated with only *B. bronchiseptica* (Bb/NV/NCh), with a group average of 1.8% of the lung affected (Figure 2). There was not a significant increase in the percentage of gross pneumonia in the NV/Ch group when compared to the control group, though the percentage of pigs in each group presenting with lesions was different (100 vs. 25%, respectively). Also, there was not a significant difference between NV/Ch and LAIV/Ch groups (*p* > 0.05). The average percentage of lung affected by lesions for the NV/Ch group was 4.1 ± 2.9 compared to 2.8 ± 4.3 for the LAIV/Ch group. There was an increase in the percentage of lung affected in the Bb/NV/Ch and Bb/LAIV/Ch groups when compared to the strict control group (*p* < 0.05), but not between the Bb/NV/Ch and Bb/LAIV/Ch groups (*p* > 0.05).

**Figure 3:**
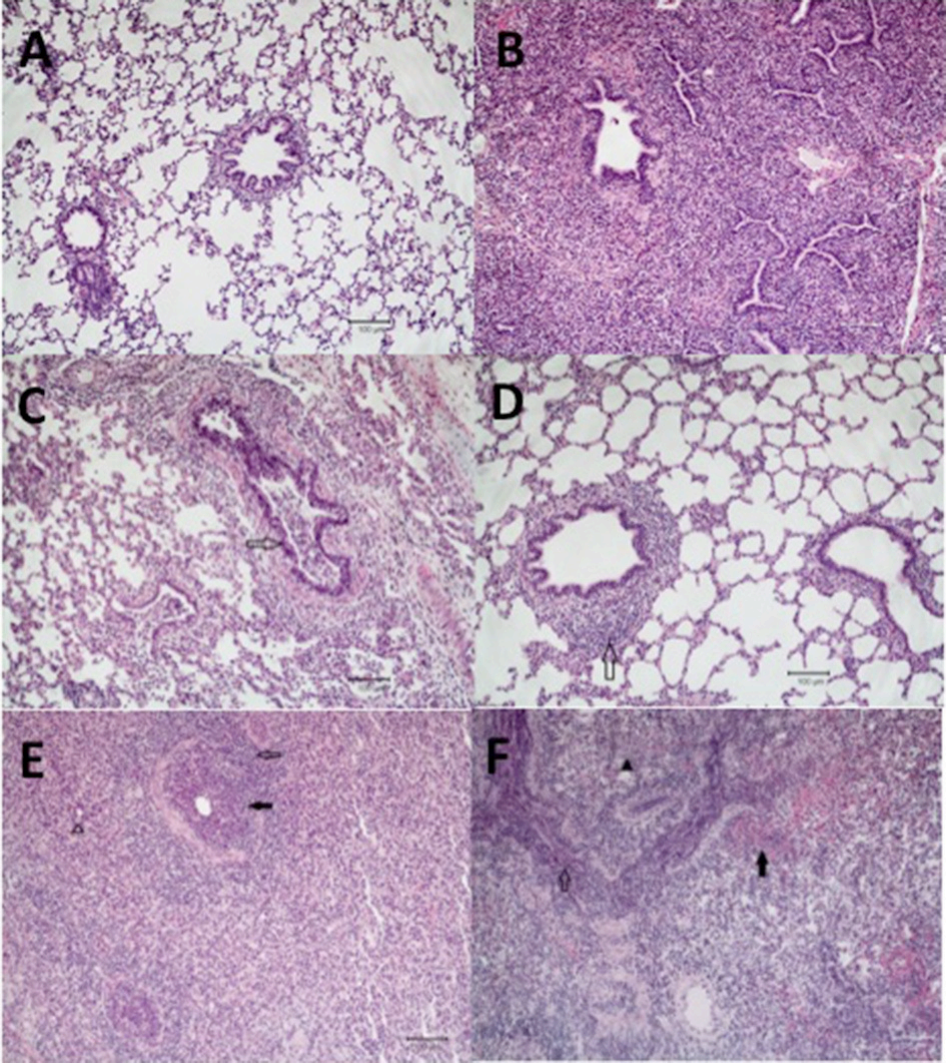
Microscopic lung lesions from representative pigs at necropsy. Groups of pigs were inoculated with *B. bronchiseptica* (Bb) on day−7, LAIV on days 0 and 21, and subsequently challenged (Ch) with heterologous IAV on day 42 as described in materials and methods. A Non-vaccinated (NV) group and a NV/non-challenged (NV/NCh) control group were included as controls. At 5 days post-infection lung sections were collected at necropsy and processed as described in materials and methods. Photomicrographs showing **(A)** normal lung in a non-infected control pig (NV/NCh); **(B)** interstitial thickening due to fibroplasia with mononuclear alveolar infiltrates in a pig chronically infected with Bb-only (Bb/NV/NCh); **(C)** suppurative bronchiolitis with epithelial necrosis (open arrow), characterized by loss of cilia and nuclear pyknosis, from a pig infected with IAV-only (NV/Ch); **(D)** peribronchiolar lymphocyte infiltration and BALT hyperplasia (open arrow) in a vaccinated pig challenged with IAV (LAIV/Ch); **(E)** suppurative bronchiolitis with epithelial necrosis (closed arrow), submucosal lymphohistiocytic inflammation, peribronchiolar lymphocytic infiltration (open arrow), interstitial pneumonia, and alveoli filled with neutrophils (open arrow head) demonstrating lesions of IAV infection and acute Bordetellosis in a pig that was infected with Bb and subsequently challenged with IAV (Bb/NV/Ch); and **(F)** alveoli and bronchioles filled with neutrophils and mononuclear cells (open arrow), alveoli with areas of epithelial necrosis and hemorrhage (closed arrow), and interstitial pneumonia (closed arrow head) demonstrating lesions of acute Bordetellosis in a vaccinated pig that had been infected with Bb and subsequently challenged with IAV (Bb/LAIV/Ch). Macropscopic pneumonia score for pig in which representative sections were selected are indicated with a red data point in Figure 2.

Microscopic lesions were either not present or minimal (limited to mild interstitial thickening) in the strict control group (NV/NCh; Figure 3A), as well as in all but 2 of the pigs inoculated with *B. bronchiseptica* alone (Bb/NV/NCh). The two Bb/NV/NCh pigs with microscopic changes had lesions consistent with chronic *B. bronchiseptica* pneumonia characterized by moderate thickening of the alveolar septa with fibrin and collagen, type II pneumocyte hyperplasia, and alveolar spaces variably filled with macrophages (30) (Figure 3B). Pigs inoculated with IAV alone (NV/Ch) had mild lesions consistent with IAV infection characterized primarily by suppurative bronchitis and bronchiolitis with epithelial necrosis and peribronchiolar lymphocytic infiltration (31) (Figure 3C). The presence and severity of interstitial pneumonia was minimal to mild in the NV/Ch group. The IAV-associated lesions were diminished in the vaccinated group (LAIV/Ch) when compared to the non-vaccinated group (NV/Ch). In particular the suppurative bronchitis or bronchiolitis with epithelial necrosis was reduced; however, there was peribronchiolar lymphocyte infiltration and bronchus associated lymphoid tissue (BALT) hyperplasia in the majority of LAIV/Ch pigs (Figure 3D).

Pigs that were infected with *B. bronchiseptica* and subsequently challenged with IAV (Bb/NV/Ch) had microscopic lesions consistent with both IAV infection as well as acute and chronic *B. bronchiseptica* pneumonia (Figure 3E). Influenza lesions included suppurative bronchitis and bronchiolitis with epithelial necrosis and submucosal lymphohistiocytic inflammation, as well as peribronchiolar lymphocytic infiltration. However, the suppurative bronchitis and bronchiolitis tended to be more severe than that observed in pigs infected with IAV alone, and furthermore alveoli were variably filled with neutrophils and macrophages with areas of alveolar epithelial necrosis, hemorrhage, and type II pneumocyte hyperplasia, which is consistent with acute Bordetellosis. In addition, in sections from some Bb/NV/Ch pigs there were areas consistent with chronic Bordetellosis characterized by interstitial pneumonia consisting of alveolar septal thickening with mononuclear cells as well as fibrin and collagen (Figure 3E).

Finally, as noted in the LAIV/Ch group, vaccinated pigs that had been infected with *Bordetella* and challenged with IAV (Bb/LAIV/Ch) had diminished bronchial and bronchiolar epithelial necrosis. However, these pigs had lesions consistent with both acute and chronic *Bordetella* pneumonia, including acute lesions consisting of alveoli and bronchioles that were variably filled with neutrophils and/or macrophages and alveoli with areas of epithelial necrosis, hemorrhage, and type II pneumocyte hyperplasia (Figure 3F). Sections from some of the pigs in Bb/LAIV/Ch group also contained chronic lesions of interstitial pneumonia consisting of alveolar septal thickening with mononuclear cells as well as fibrin and collagen, which is consistent with chronic *B. bronchiseptica* infection.

### *B. bronchiseptica* respiratory tract colonization was not affected by IAV challenge, regardless of LAIV vaccine status

**Figure 4:**
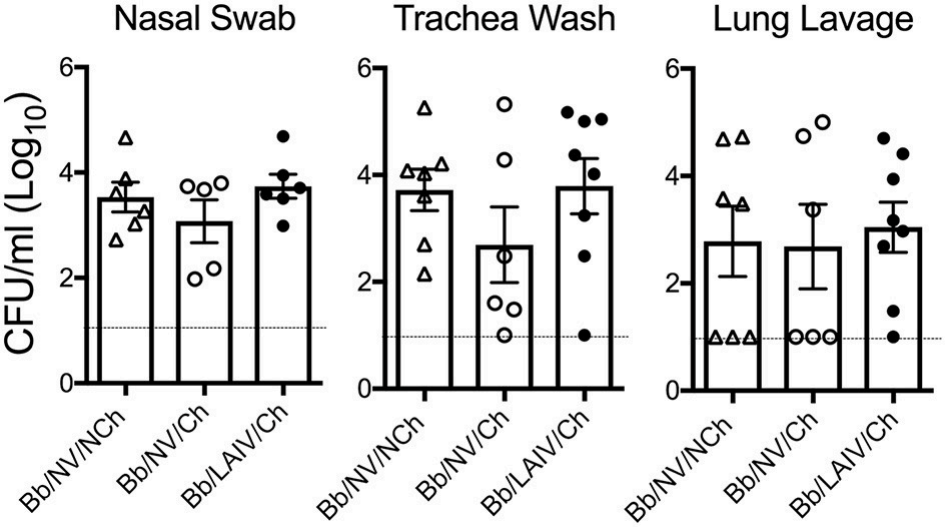
*B. bronchiseptica* respiratory tract colonization was not affected by IAV challenge, regardless of LAIV vaccine status. Groups of pigs were inoculated with *B. bronchiseptica* (Bb) on day−7, LAIV on days 0 and 21, and subsequently challenged (Ch) with heterologous IAV as described in materials and methods. At 5 days post-infection, nasal swab, trachea wash and lung lavage were collected from each pig at necropsy and Bb enumerated to determine if IAV effected Bb colonization in the respiratory tract. Each dot represents a single animal in that respective treatment group, with the average ± SEM indicated. The dotted line represents the limit of detection. Data was analyzed using a one-way ANOVA with a Tukey's post-test (GraphPad Prism 6). There were no significant differences between groups (*p* > 0.05).

To determine if *B. bronchiseptica* colonization or levels in the respiratory tract were altered following IAV challenge, the amount of *B. bronchiseptica* in nasal swab, trachea wash, and lung lavage collected from each pig at necropsy was determined. *B. bronchiseptica* was recovered from 100% of the nasal swabs collected on 5 dpi from pigs inoculated with *B. bronchiseptica* and there was no significant difference in colonization levels between the groups (*p* > 0.05, Figure 4). *B. bronchiseptica* colonization in the trachea and lung were also similar between groups, with no significant differences noted (*p* > 0.05, Figure 4). *B. bronchiseptica* was not recovered from any sample collected from pigs not inoculated with *B. bronchiseptica* (data not shown).

### LAIV vaccine efficacy following intranasal heterologous IAV challenge

Experimental intranasal LAIV vaccination has been shown to provide significant protection against heterologous IAV replication following challenge, including protection against the challenge virus used in this study (8). To determine if *B. bronchiseptica* colonization altered LAIV vaccine efficacy, IAV titers in the respiratory tract following heterologous IAV challenge were determined.

**Figure 5:**
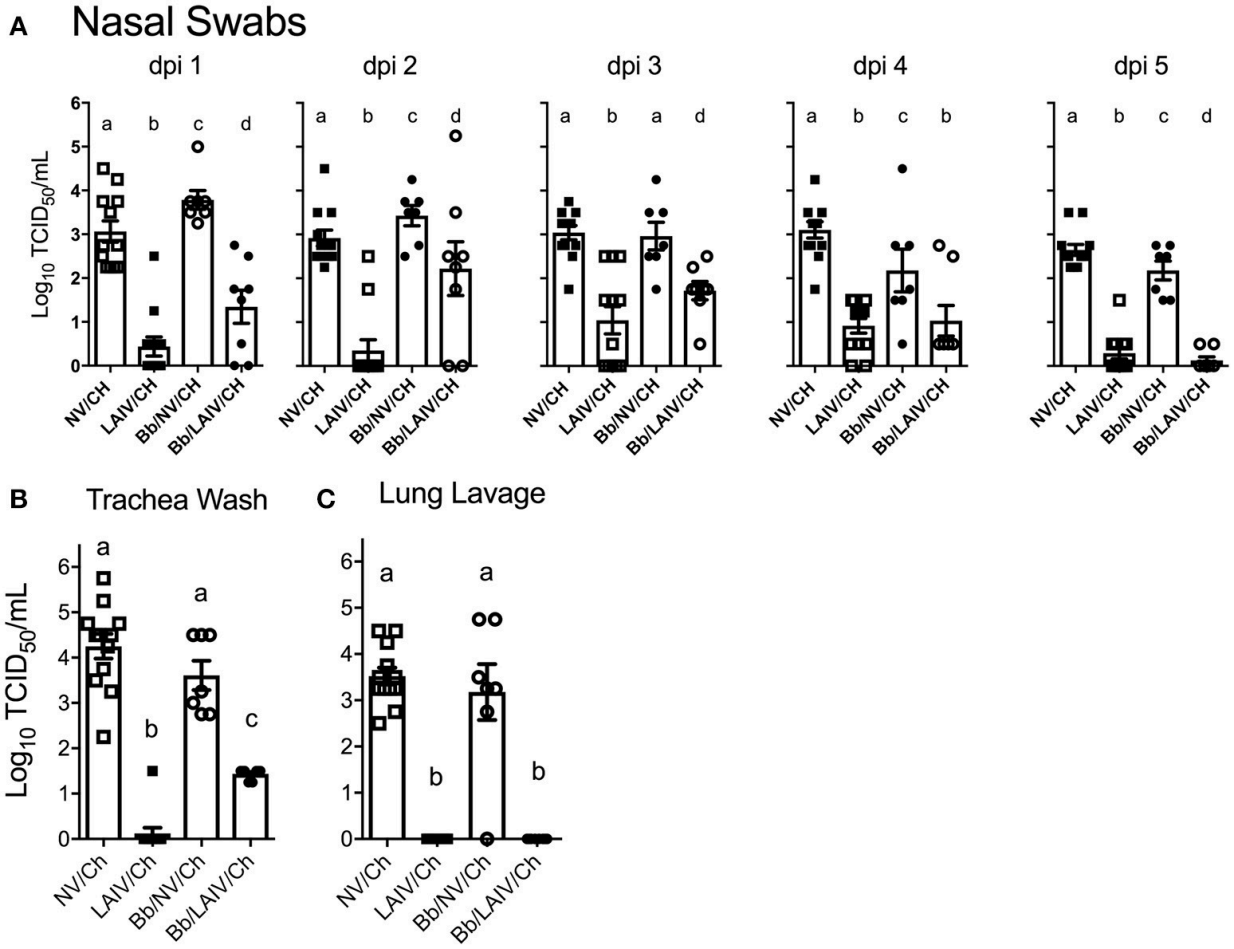
IAV titers in the respiratory tract were affected by *B. bronchiseptica* colonization. Groups of pigs were inoculated with *B. bronchiseptica* (Bb) on day−7, LAIV on days 0 and 21, and subsequently challenged (Ch) with heterologous IAV as described in materials and methods. **(A)** At 1–5 days post-infection (dpi), nasal swabs were collected to determine the amount of IAV being shed from the nasal cavity; **(B)** At 5 dpi trachea wash and **(C)** lung lavage were collected to determine the amount of IAV in the lower respiratory tract. Each dot represents a single animal in that respective treatment group with mean ± SEM shown and was analyzed using a one-way ANOVA with a Tukey's post-test (GraphPad Prism 6). Groups with different letter designations were significantly different (*p* < 0.05).

Nasal swabs were collected on the day of IAV challenge and daily through 5 dpi. Table 2 reports the number of pigs in each group shedding virus and Figure 5A reports titers for each animal and the average titer (log_10_ TCID_50_/ml) for the indicated group for each indicated dpi. One day after intranasal IAV challenge, 100% of the NV/Ch pigs were shedding virus with an average IAV titer (log_10_ TCID_50_/ml) of 3.06 ± 0.84, which was significantly increased over the 0.44 ± 0.75 for the LAIV/Ch group (*p* < 0.05). The significant difference in IAV titers in nasal swabs between NV/Ch and LAIV/Ch group was noted for days 2-5 as well (Figure 5A). There was a significant difference in IAV titers in nasal swabs between Bb/NV/Ch and Bb/LAIV/Ch groups at every time point as well (*p* < 0.05), indicating that *B. bronchiseptica* colonization did not completely inhibit efficacy of the LAIV vaccine. Plotting of nasal swab IAV titers for each individual animal in Figure 5A indicated a range in the amount of virus shed from a pig in each respective group. Thus, while average nasal swab titers were significantly different between groups, there was a broad range in shedding titers within a group.

**Table 2:** Percent of pigs in each group positive for IAV in nasal swabs on the indicated day post-infection.

**Treatment Group^a^**	**Day post-infection**				
	**1**	**2**	**3**	**4**	**5**
NV/Ch	100% (12/12)^b^	100% (12/12)	100% (12/12)	100% (12/12)	100% (12/12)
LAIV/Ch	42% (5/12)	42% (5/12)*	58% (7/12)*	83% (10/12)	42% (5/12)
Bb/NV/Ch	100% (7/7)	100% (7/7)	100% (7/7)	100% (7/7)	100% (7/7)
Bb/LAIV/Ch	75% (6/8)	100% (8/8)	100% (8/8)	88% (7/8)	25% (2/8)

a *NV, Non-Vaccinated; Ch, Challenged; LAIV, Live-Attenuated Influenza Virus; Bb, B. bronchiseptica*.

b Percent positive in group, number of positive samples over total number of samples in respective group.

*Fishers exact test p < 0.05*.

IAV nasal shedding was greater in the Bb/NV/Ch group compared to NV/Ch group at dpi 1 and 2, no different at dpi 3, and by dpi 4 and 5, titers were greater in NV/Ch group (Figure 5A). Similarly, IAV shedding was greater in the Bb/LAIV/Ch group compared to LAIV/Ch on days 1–3, no different on day 4, and by dpi 5 titers were greater in the LAIV/Ch pigs (Figure 5A). However, on dpi 5 the average amount of virus shed from the LAIV vaccinated group, regardless of *B. bronchiseptica* status, was less than 0.5 log_10_ TCID_50_/ml indicative of LAIV vaccine efficacy. All of the pigs (100%) in the Bb/LAIV/Ch group were shedding virus on days 2 and 3, but only 42 and 58% of the pigs in LAIV/Ch group were shedding, respectively, but by dpi 4 and 5 there was not a significant difference in the percentage of pigs in the Bb/LAIV/Ch and LAIV/Ch groups shedding virus (Table 2). These results indicate that *B. bronchiseptica* infection, regardless of LAIV vaccination status, affected IAV replication in the nasal cavity.

IAV titers in the lower respiratory tract on dpi 5 were also evaluated, and there was a significant difference (*p* < 0.05) in the titer of IAV in the trachea wash of LAIV/Ch pigs when compared to Bb/LAIV/Ch pigs. Specifically, IAV was isolated from the trachea wash of 1 of 12 pigs in the LAIV/Ch group whereas IAV was isolated from the trachea wash of all pigs (8/8) in the Bb/LAIV/Ch group, and there was a significant difference in titers between the two treatment groups (*p* < 0.05; Figure 5B). However, *B. bronchiseptica* did not completely interfere with LAIV efficacy, as the titer of IAV in the trachea wash from Bb/LAIV/Ch group was significantly reduced compared to the Bb/NV/Ch group (*p* < 0.05). As expected, the average titer of IAV in the trachea and lung lavage from pigs in the LAIV/Ch group was significantly reduced compared to the NV/Ch group, indicative of the protection against heterologous virus replication following LAIV vaccination (Figures 5B,C). Protection against heterologous virus replication was also noted in the Bb/LAIV/Ch group, as IAV was not isolated from the lung lavage of any pig in this group.

### *B. bronchiseptica* colonization did not alter the development of IAV-specific mucosal IGA following LAIV administration

**Figure 6:**
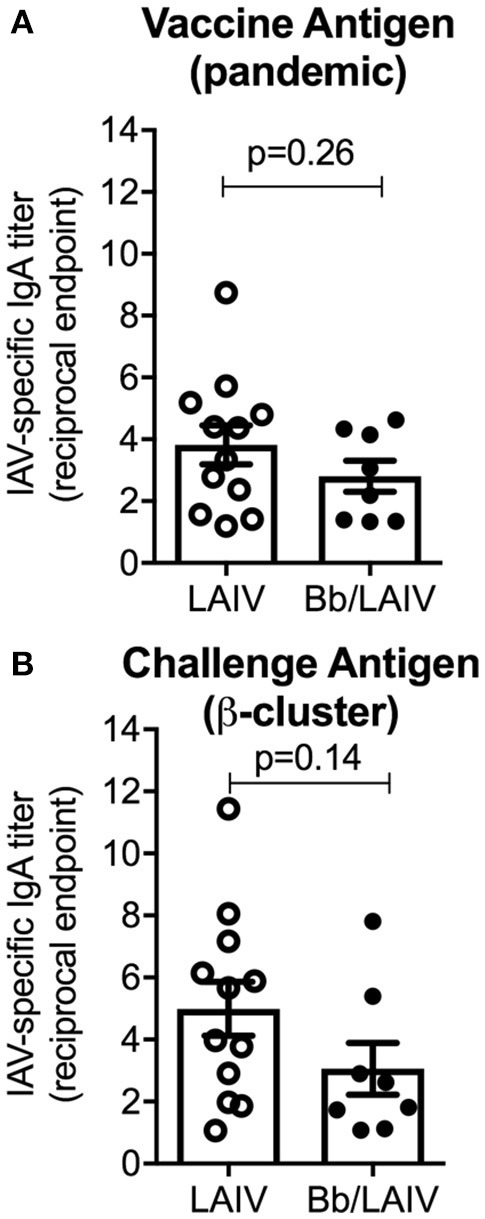
*B. bronchiseptica* colonization did not alter the development of IAV-specific mucosal IgA following LAIV administration. Groups of pigs were inoculated with *B. bronchiseptica* (Bb) on day−7, LAIV on days 0 and 21, and nasal wash collected on day 42 as described in materials and methods. Titers of IgA in nasal wash specific to **(A)** vaccine virus and **(B)** challenge virus were determined using a whole virus ELISA to evaluate the impact of Bb on LAIV immunogenicity. Data was analyzed using an unpaired students *t*-test (GraphPad Prism 6) and there were no significant differences between groups (*p* > 0.05).

To determine if *B. bronchiseptica* colonization inhibited LAIV immunogenicity, IgA titers to IAV in nasal wash samples collected immediately prior to IAV challenge were determined (Figure 6). Reciprocal endpoint titers of IgA to both vaccine virus and challenge virus were determined using a whole virus ELISA. There was no significant difference in nasal wash IgA titers to either virus between the two vaccinated group (*p* > 0.05) indicating that LAIV immunogenicity in the nasal cavity was not negatively impacted by *B. bronchiseptica* colonization. A Pearson's correlation test evaluating NW MN/10 IAV-specific IgA reciprocal titers reported in Figure 6 to IAV nasal swab titers (average over 3 days and 5 days) reported in Figure 5A was performed, but there was not statistical correlation between the data (data not shown).

### Lung cytokine levels were primarily associated with IAV challenge of non-vaccinated pigs, regardless of *B. bronchiseptica* colonization

**Figure 7:**
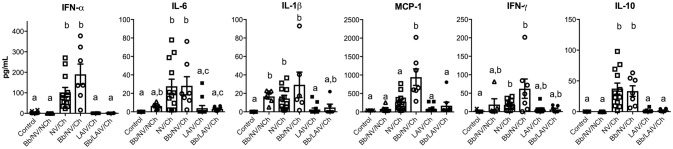
Altered cytokine levels in the lung were associated with IAV challenge on non-vaccinated pigs, regardless of *B. bronchiseptica* colonization. Groups of pigs were inoculated with *B. bronchiseptica* (Bb) on day−7, LAIV on days 0 and 21, and subsequently challenged (Ch) with heterologous IAV as described in materials and methods. On 5 days post-infection lung lavage was collected and levels of indicated cytokine measured by multiplex ELISA. Each dot represents a single animal in that respective treatment group with mean ± SEM shown and was analyzed using a Kruskal-Wallis test with a Dunn's multiple comparisons post-test (GraphPad Prism 6). Groups with different letter designations were significantly different (*p* < 0.05).

To determine if lung lesion severity was associated with an inflammatory cytokine response, the levels of several cytokines were measured in the lung lavage collected at necropsy (Figure 7). IFN-α, an antiviral cytokine, was increased in the lavage of non-vaccinated pigs challenged with IAV, regardless of *B. bronchiseptica* colonization (*p* < 0.05). This was also observed for IL-6 and IL-10 (*p* < 0.05). Specifically, cytokine levels were increased in NV/Ch and Bb/NV/Ch pigs over respective vaccine groups (LAIV/Ch and Bb/LAIV/Ch) for all tested cytokines but MCP-1. MCP-1 levels were significantly increased in non-vaccinated group that were colonized with Bb and challenged with IAV over non-vaccinated and IAV infected pigs, suggesting Bb co-infection led to enhanced MCP-1 production. IL-1β levels were increased in lungs of Bb/NV/NCh pigs over control pigs; however, IL-1β levels in Bb/NV/NCh pigs were not significantly different than levels of the other *B. bronchiseptica* colonized groups (Bb/NV/NCh and Bb/LAIV/Ch). While there was a significant increase in IFN-γ levels in the lung following IAV challenge of non-vaccinated pigs, levels were not significantly different than those in vaccinated and challenged pigs, regardless of Bb status. Collectively, these data indicate cytokine levels were increased primarily in pigs that had been challenged with IAV, but not previously vaccinated.

## Discussion

Intranasally delivered LAIV vaccines have been shown experimentally to provide significant cross-protection against heterologous IAV in pigs (7, 8, 32–36). The majority, if not all, of experimental challenge studies in swine with LAIV use pigs that have been procured from high health status herds that are free of many of the bacteria that can be pathogenic under some conditions (i.e., pathobiont) and associated with secondary disease in commercial settings. These bacteria include, but are not limited to, *Bordetella, Mycoplasma, Haemophilus* and *Streptococcus*, which are also associated with the respiratory disease complex, a multifactorial disease in which infectious agents, environment, and management practices play a role in susceptibility (37). Much like *B. pertussis* in humans, *B. bronchiseptica* in pigs can be isolated from the respiratory tract without evidence of pathology or clinical disease. However, *B. bronchiseptica* has been shown to cause significant disease when pigs are co-infected with other respiratory viruses, including IAV (17, 18). Inflammation resulting from epithelial and immune cell changes following IAV infection suppresses antibacterial immune mechanisms such that bacterial dynamics and infection severity is impacted [reviewed in Smith and Mccullers (38), Robinson et al. (39)]. LAIV vaccination provides protection against IAV infection, which results in a reduction of secondary bacterial pneumonia (40, 41) and may limit antibiotic usage. However, intranasal administration of LAIV vaccine can alter commensal replication and colonization, though these changes are limited to the upper respiratory tract (23, 42, 43). In the current study, LAIV administration had minimal effect, if any, on *B. bronchiseptica* density in the upper respiratory tract and we did not appreciate any signs of clinical disease following LAIV administration (data not shown). At day 42 after LAIV administration, which was 49 days after Bb inoculation, there was a significant difference in the amount of Bb in the nasal passages of Bb/NV and Bb/LAIV pigs. However, this could be due to commonly measured shifts in Bb nasal colonization around 7 weeks post-inoculation (25, 26), which is not uncommon. Given that no other changes in Bb colonization following LAIV administration were detected, we do not expect the difference was due to LAIV administration, though it cannot be completely ruled out. It is possible that LAIV administration altered the overall bacterial community structure in the upper respiratory tract of pigs, though additional studies are needed to assess such changes.

The IAV strain used for challenge in this study was not an exact match to the vaccine antigen, as viruses in the β-cluster lineage (based on HA genetic and antigenic characteristics) are distantly related to pdm-lineage viruses (44–46). Thus, as previously shown (8), we anticipated some replication of the challenge virus even in the LAIV vaccinated groups but limited detection in the lower respiratory tract by dpi 5. The presence of lung lesions associated with IAV infection of LAIV vaccinates provided support that IAV replicated in the lower respiratory tract of LAIV/Ch pigs, though there was significant protection against virus replication and pathology. There was not a significant increase in cytokine levels in the lungs of LAIV/Ch pigs compared to control pigs, and levels of IFN-α, IL-1β, and IL-6 in LAIV/Ch pigs were reduced compared to NV/Ch pigs providing support that IAV replication in the lower respiratory tract was readily controlled by LAIV-induced immunity.

Overall *B. bronchiseptica* colonization did not interfere with LAIV immunogenicity, but LAIV vaccination did not necessarily prevent the negative impact of IAV/*B. bronchiseptica* co-infection. Although *B. bronchiseptica* encodes a number of immunomodulatory proteins (47–49), its presence at the time of LAIV administration did not impair induction of mucosal IgA to IAV. We expect LAIV induced the production of mucosal IAV-specific T cells, which has been shown in other species (50); but, the impact of *B. bronchiseptica* colonization on IAV-specific T cell development is unknown. Although IAV-specific IgA was induced in the upper respiratory tract of pigs following LAIV vaccination during *B. bronchiseptica* colonization, it was unable to provide full protection upon IAV challenge in the face of *B. bronchiseptica* infection. Thus, LAIV vaccine based immunity could not protect against the negative interaction that occurred with IAV/*B. bronchiseptica* co-infection. *B. bronchiseptica* has a sophisticated environmental sensing system (Bvg locus) that drives expression of numerous virulence factors (51), including unique regulatory system controlling expression in the lower respiratory tract (52). There was minimal pathology in the lungs on Bb-only pigs (Bb/NV/NCh), and lesions present were characteristic of chronic *B. bronchiseptica* infection, suggesting that virulence gene expression so long after initial *B. bronchiseptica* inoculation was minimal. However, environmental changes in the respiratory tract associated with IAV infection, such as epithelial damage and/or altered abundance of soluble immune mediators (cytokines, defensins), or leakage of serum proteins into the airway may have subsequently altered *B. bronchiseptica* gene expression such that cytotoxic factors were produced. *Bordetella* exposure to albumin enhances production of adenylate cyclase toxin (ACT) (53), and ACT disrupts bronchial epithelial integrity (54). Enhanced expression of *B. bronchiseptica* virulence factors likely contributed to the appearance of lesions associated with acute *B. bronchiseptica* infection. The limited replication of challenge virus in LAIV vaccinates also colonized with *B. bronchiseptica* (Bb/LAIV/Ch) appeared to be enough to incite pulmonary lesions commonly associated with acute *B. bronchiseptica* infection. Lesion characteristics associated with acute *B. bronchiseptica* infection were noted in both Bb/NV/Ch and Bb/LAIV/Ch groups, but not the Bb/NV/NCh group. Pathologic changes were not due to an increase in bacterial burden (Figure 4), which has been an ascribed pathologic interaction by other commensal organisms following IAV infection (55), nor significant increases in proinflammatory cytokine levels. Only recently has *B. bronchiseptica* gene expression *in vivo* been evaluated (56), and future work aimed at identifying factors that alter gene expression may provide insight on the mechanism by which *B. bronchiseptica* plays a role in secondary disease.

Mucosal cell-mediated immune (CMI) responses play a role in IAV cross-protection (57, 58), though this was not directly assessed in the current study. *B. bronchiseptica* may have altered the induction of cell-mediated immunity in the lungs of LAIV vaccinated pigs, though this is unlikely given that IAV-specific IgA was produced and class switching to IgA requires CD4 T cell help. Histologic changes in the lungs of pigs in the LAIV/Ch and Bb/LAIV/Ch group, but not NV/Ch group, consisted of peribronchiolar lymphocyte cuffing and bronchus-associated lymphoid tissue (BALT), indicating that LAIV likely induced a CMI response in the lungs regardless of *B. bronchiseptica* colonization. Again, changes in the respiratory tract associated with IAV/*B. bronchiseptica* co-infection, regardless of vaccine status, may hinder immune-protection. It's possible that if the vaccine and challenge antigen were more closely related, such that the LAIV vaccine provided sterilizing immunity against challenge, there would not have been an induction of acute Bordetellosis in the Bb/LAIV/Ch group, though further investigation is necessary to test this hypothesis. Overall, LAIV administration provided significant protection against a distantly related strain of IAV by substantially decreasing replication of the virus in the respiratory tract. Thus, the data presented do not negate the efficacy of LAIV vaccination, but instead indicate that controlling *B. bronchiseptica* colonization in swine could limit the negative interaction between IAV and *Bordetella* on swine health.

## Ethics statement

This study was carried out in accordance with the recommendations of the USDA-ARS-National Animal Disease Center Animal Care and Use Committee and the protocol was approved by the Committee.

## Author contributions

HH, SB, and CL designed and executed the animal studies, analyzed data, and wrote the manuscript.

### Conflict of interest statement

HH was funded through an agreement with Boehringer Ingelheim Vetmedica, Inc. The remaining authors declare that the research was conducted in the absence of any commercial or financial relationships that could be construed as a potential conflict of interest.
